# Life-Threatening Sochi Virus Infections, Russia

**DOI:** 10.3201/eid2112.150891

**Published:** 2015-12

**Authors:** Detlev H. Kruger, Evgeniy A. Tkachenko, Vyacheslav G. Morozov, Yulia V. Yunicheva, Olga M. Pilikova, Gennadiy Malkin, Aydar A. Ishmukhametov, Patrick Heinemann, Peter T. Witkowski, Boris Klempa, Tamara K. Dzagurova

**Affiliations:** Charité School of Medicine, Berlin, Germany (D.H. Kruger, P. Heinemann, P.T. Witkowski, B. Klempa);; Chumakov Institute of Poliomyelitis and Viral Encephalitides, Moscow, Russia (E.A. Tkachenko, G. Malkin, A.A. Ishmukhametov, T.K. Dzagurova);; Medical State University, Samara, Russia (V.G. Morozov);; Anti-Plague Stations, Sochi, Russia (Y.V. Yunicheva);; Anti-Plague Stations, Novorossiysk, Russia (O.M. Pilikova);; Slovak Academy of Sciences, Bratislava, Slovakia (B. Klempa)

**Keywords:** Hantavirus, hemorrhagic fever with renal syndrome, Apodemus ponticus, Sochi virus, Russia, viruses

## Abstract

Sochi virus was recently identified as a new hantavirus genotype carried by the Black Sea field mouse, *Apodemus ponticus*. We evaluated 62 patients in Russia with Sochi virus infection. Most clinical cases were severe, and the case-fatality rate was as high as 14.5%.

Hantaviruses are zoonotic pathogens transmitted from small animals to humans. Hantavirus disease in the Americas is called hantavirus pulmonary syndrome and in Asia and Europe is called hemorrhagic fever with renal syndrome (HFRS). Both syndromes can lead to cardiopulmonary and renal failure (*1*). Recently we described a new hantavirus, Sochi virus, from the administrative region Krasnodar (including the city of Sochi), southern European Russia, which was isolated in cell culture from a Black Sea field mouse (*Apodemus ponticus*) and a patient with fulminant hantavirus disease who died of shock and combined kidney and lung failure (*2*–*4*). Molecular taxonomical analyses identified Sochi virus as a new genotype within the Dobrava-Belgrade virus (DOBV) species (*5*). Here we show that HFRS caused by Sochi virus infection occurs in the geographic region where *A. ponticus* mice are prevalent. For 62 patients infected by this virus during 2000–2013, we evaluated clinical and epidemiologic data.

## The Study

Serum of patients with suspected acute hantavirus disease from the Krasnodar region were screened for hantavirus antibodies by indirect immunofluorescence assays and ELISA. Sixty-two patients showed clear DOBV IgG seropositivity. During the acute phase of illness, all patients tested positive for DOBV IgM (data not shown). For 26 patients, sufficient volumes of follow-up serum were available for additional focus reduction neutralization assays to specify neutralizing antibodies. All serum samples exhibited substantially higher neutralizing titers toward DOBV than toward Puumala virus, Hantaan virus, and Seoul virus. When the neutralizing effect of DOBV-positive patients’ serum were compared against the different human pathogenic genotypes of DOBV (Dobrava, Kurkino, and Sochi), all serum predominantly reacted with the Sochi genotype (Technical Appendix Table 1).

We successfully obtained virus genomic large (L) segment sequences from 2 patients (no. 51, specimen no. 6882; no. 59, specimen no. 10752). In the neighborhood of the residence of patient no. 59, mice were trapped, and hantaviral L and small (S) segment regions from 2 *A. ponticus* animals (specimen nos. 10636, 10645) were amplified. The sequences obtained were deposited in GenBank under accession nos. KM192207–09 and KP878308–10 (L segment) and KP878311–13 (S segment) (Technical Appendix Table 2). Samples from virus-positive mice were phylogenetically characterized by analysis of a 242-bp region of their *cytB* gene; all of them clustered with those of the previously identified *A. ponticus* animals (*3*) (data not shown). In addition, the *A. ponticus*–derived isolate Sochi/Ap (*4*), the patient-derived isolate Sochi/hu (*5*), an S segment sequence from a mouse (GK/Ap) trapped near the home of the previously described Krasnodar patient (*4*), and sequences originating from 2 *A. ponticus* mice sampled near the Black Sea coast, 43/Ap and 79/Ap, were included in the molecular analyses of the virus.

**Figure 1:**
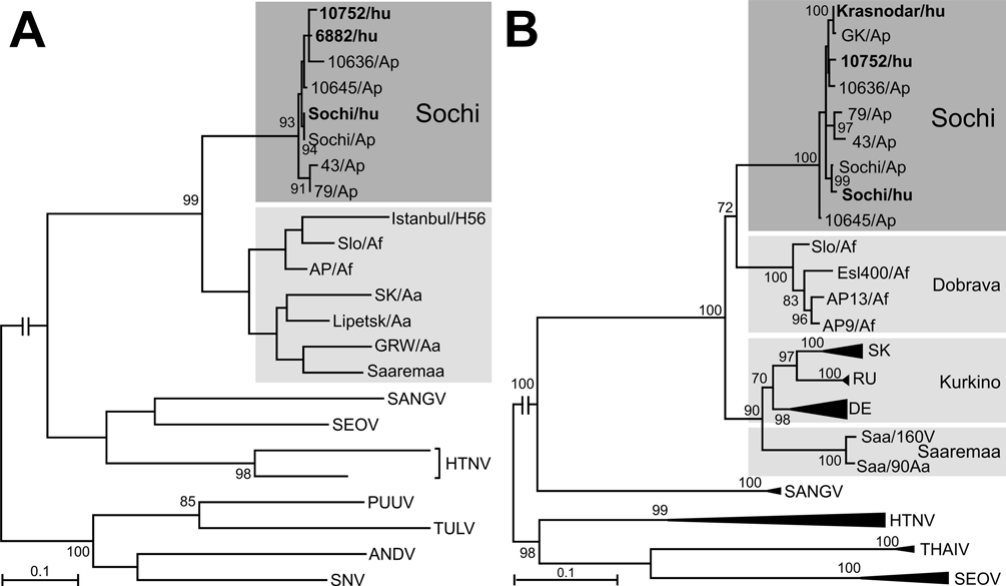
Phylogenetic analysis segment sequences of Sochi virus, Russia: A) 347-bp large (L) segment sequence; B) 1,197-bp small (S) segment sequence. Virus sequences derived from patients (shown in bold type) and *Apodemus ponticus* mice cluster within the Sochi genotype of DOBV. Evolutionary analysis was conducted in MEGA6 (*6*). The evolutionary history was inferred by using the maximum-likelihood method based on the Tamura 3-parameter model with a discrete gamma distribution and 5 rate categories (analysis in panel A) and on the general time reversible model with gamma rates and heterogeneous patterns (analysis in panel B), respectively, which were estimated to be the best-fit substitution model according to the Bayesian information criterion. Scale bars indicate an evolutionary distance of 0.1 substitutions per position in the sequence. Bootstrap values >70%, calculated from 500 replicates, are shown at the tree branches. GenBank accession numbers of all sequences used in the analysis are listed in Technical Appendix Table 1). Dark gray shading iindicates cluster of DOBV-Sochi strains; light gray shading indicates different clusters of strains from other DOBV genotypes. ANDV, Andes virus; DOBV, Dobrava-Belgrade virus; HTNV, Hantaan virus; PUUV, Puumala virus; SANGV, Sangassou virus; SEOV, Seoul virus; SNV, Sin Nombre virus; THAIV, Thailand virus; TULV, Tula virus.

The patient-derived sequences 6882/hu, 10752/hu, and Sochi/hu clearly cluster with *A. ponticus*–derived sequences 43/Ap, 79/Ap, 10636/Ap, 10645/Ap, and Sochi/Ap (Figure 1, panel A). In the analysis of the S segment, we obtained a very similar result; the patient-derived sequences 10752/hu, Krasnodar/hu, and Sochi/hu cluster with *A. ponticus*–associated sequences 43/Ap, 79/Ap, 10636/Ap, 10645/Ap, GK/Ap, and Sochi/Ap (Figure 1, panel B). In analysis of both L and S segments, the Sochi virus strains form a unique group, clearly distinguishable from all other DOBV genotypes.

**Figure 2:**
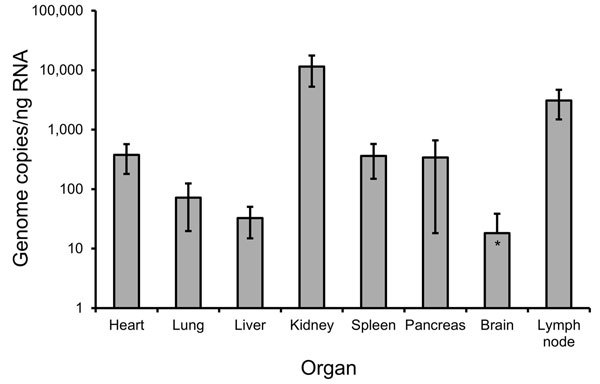
Quantification of hantavirus RNA in tissue biopsies from a 50-year-old Sochi virus–infected man (patient no. 59), Russia. Two independent approaches were performed to extract RNA from each organ. Quantitative reverse transcription PCR previously developed for DOBV (*7*) was used to measure virus load in the analyzed biopsy samples. Three quantitative reverse transcription PCR estimations were conducted for every RNA extraction, followed by calculation of mean values and SDs. Viral RNA levels are shown as genome copies per nanogram of total RNA isolated from the samples. Error bars indicate SD.

Specimens from different organs of deceased patient no. 59 were analyzed for virus load. The highest concentration was detected in kidney (11,446 copies/ng RNA) and lymph node (3,086 copies/ng RNA), whereas the least virus RNA (10–100 copies/ng RNA) was detected in lung, brain, and liver (Figure 2).

The clinical disease severity of the 62 Sochi virus–infected patients investigated (Table 1) was subdivided into mild, moderate, or severe following the standard Russian criteria (i.e., length of febrile phase, minimal blood pressure in the hypotonic phase, extent of hemorrhagic symptoms, minimal urine production, serum creatinine level, and extent of proteinuria) (Technical Appendix Table 3). The case-fatality rate (CFR) was as high as 14.5% (9/62 patients). Including fatalities, severe disease developed in nearly 60% of patients, whereas the remaining 40% of cases were moderate. The average age of all patients was 33 years. A significantly higher proportion of patients were males (p = 1.05 × 10^–9^). Moreover, severe disease developed in most affected male patients (66.7%) but in only 35.7% of affected female patients (p = 0.037). The fact that only 2 of 9 fatal cases occurred in female patients (Table 1) underscores this finding.

**Table 1:** Comparisons in clinical outcome, age, and sex of 62 patients with Sochi virus infection, Russia*

Characteristic	Total			Sex, no. (%)			Age, y, n/N (%)	
	No. (%)	Median age, y (range)		M, n = 48	F, n = 14		7–15	>15
No. patients	62 (100)	33.3 (7–57)		**48 (77.4)**	**14 (22.6)**		**6/62 (9.7%)**	**56/62 (90.3)**
Outcome								
Died	9 (14.5)	38.6 (19–53)		7 (14.6)	2 (14.3)		0/6	9/56 (16.1)
Survived	53 (85.5)	32.4 (7–57)		41 (85.4)	12 (85.7)		6/6 (100)	47/56 (83.9)
Illness course								
Severe, including fatal	37 (59.7)	33.1 (10–57)		**32 (66.7)**	**5 (35.7)**		3/6 (50)	34/56 (60.7)
Moderate, mild	25 (40.3)	33.6 (7–57)		**16 (33.3)**	**9 (64.3)**		3/6 (50)	22/56 (39.3)

*Bold type indicates statistically significant differences between sex or age groups. Comparison of binomial population proportions analysis as implemented in Statlets (NWP Associates, Inc., http://www.mrs.umn.edu/~sungurea/statlets/statlets.htm) indicates rejection of the null hypothesis (claiming that the 2 proportions are equal) at significance level of p<0.05.

All 9 patients with fatal infections died of multiorgan failure and shock (Table 2). Postmortem examination showed multiple hemorrhages and edema in internal organs, including kidneys and lungs. The patients died within 8.2 days (range 3–16 days) after disease onset. An extraordinary fulminant course was observed for patient no. 47, who died 3 days after onset and before he could be hospitalized. This 19-year-old man was the son-in-law of patient no. 48, who also died after Sochi virus infection. Both men lived at the same rural address, and rodent contact during work in haystacks was reported.

**Table 2:** Characteristics of 9 deceased patients with Sochi virus infection, Russia*

Patient no.	Age, y/sex	Hospitalized, no. d after onset	GI symptoms	Max serum creatinine, μmol/L†	Min platelet count, × 10^9^/L‡	Died, no. d after onset	Clinical and postmortem findings
23	33/M	5	No	148	70	8	Pneumonia; renal, cardiovascular, multiorgan failure; multiple internal hemorrhages, edema
29	29/M	Same day	Yes	282	115	6	Renal, cardiovascular, multiorgan failure; multiple internal hemorrhages, edema
30	47/F	5	Yes	391	38	12	Renal, lung failure; shock; coagulation disturbance; hemorrhagic gastroenteritis; multiple internal hemorrhages, edema
34	53/M	3	Yes	250	110	10	Multiorgan failure; coagulation disturbances; multiple internal hemorrhages
42	30/M	14	Yes	186	67	16	Uremic coma; multiorgan failure; multiple internal hemorrhages
47§	41/M	Died before hospitalization	Yes	NR	NR	3	Renal failure; multiple internal hemorrhages, edema
48§	19/M	4	Yes	192	54	6	Renal, cardiovascular failure; RDS, DIC syndrome; bleedings in pituitary, adrenal gland, intestinum, etc.
56	35/F	4	Yes	410	49	6	Cardiovascular, renal, lung, liver failure; renal tubular necrosis; lung, brain edema
59	50/M	5	Yes	310	3	7	Renal,cardiovascular failure; RDS; multiple internal hemorrhages; pleurorrhea; lung, brain edema

*DIC, disseminated intravascular coagulation; GI, gastrointestinal; max, maximum; min, minimum; RDS, respiratory distress syndrome; NR, not reported. †Reference range <96 μmol/L for female patients, <110 μmol/L for male patients. ‡Reference range 150–400 × 10^9^/L §Patient no. 47 was the father in-law of patient no. 48; both lived in the same rural residence.

## Conclusions

We have demonstrated the occurrence of human infections by Sochi virus and studied the clinical outcome for 62 patients. This virus is carried by the Black Sea field mouse (*A. ponticus*), which occurs naturally in the Transcaucasian region between the Black and Caspian Seas, including a part of southern European Russia. In anecdotal field studies in the coast region near Sochi, *A. ponticus* was the most abundant mouse species (71% of all trapped mice were identified as *A. ponticus*); moreover, 14% of trapped *A. ponticus* mice were serologically proven to be DOBV infected (*8*). This finding indicates that DOBV is the hantavirus indigenous in this geographic area and that *A. ponticus* mice are highly relevant as a hantavirus reservoir. All evidence from the natural virus reservoir, as well as serologic and molecular diagnostics of patients’ serum, shows that the virus responsible for the infections is the DOBV genotype Sochi.

Most investigated patients found to be infected by Sochi virus exhibited a severe clinical course. With a calculated CFR of 14.5%, Sochi virus might be the most deadly hantavirus outside the Americas, where 35%–50% of hantavirus infections are fatal (*1*,*9*). Even Asian Hantaan virus is estimated to be less deadly; recent studies show CFRs of 1%–3% in China and South Korea, where Hantaan virus infections play an important role in HFRS morbidity (*10*,*11*). On the other hand, increased awareness in diagnostics, treatment, and prevention by local physicians and public health authorities is expected to improve survival rates for Sochi virus infections.

Among the related viruses of the DOBV species, Sochi virus seems to have the highest level of virulence, similar to Dobrava virus (carried by *A. flavicollis* mice), which has a CFR of up to 10%–12% (*12*,*13*). As shown in larger studies, disease caused by infection with the related Kurkino genotype (carried by the western lineage of *A. agrarius* mice) is associated with a CFR of only 0.3%–0.9% (*3*,*14*). These phylogenetically related viruses exert a quite different pathogenicity in humans.

Technical Appendix Details of serodiagnostic data of 62 Sochi virus–infected patients investigated, Russia; list of virus sequences and corresponding GenBank accession numbers used in the phylogenetic analyses; and classification criteria of clinical severity of hemorrhagic fever with renal syndrome.
