# Supplementary material for: Life-Threatening Sochi Virus Infections, Russia
Source: Emerg Infect Dis. 2015 Dec;21(12):2204–8. doi: 10.3201/eid2112.150891 (PMC4672424; doi:10.3201/eid2112.150891)
Supplement: Technical Appendix — Details of serodiagnostic data of 62 Sochi virus–infected patients investigated, Russia; list of virus sequences and corresponding GenBank accession numbers used in the phylogenetic analyses; and classification criteria of clinical severity of hemorrhagic fever with renal syndrome. [file 15-0891-Techapp-s1.pdf]

Article DOI: http://dx.doi.org/10.3201/eid2112.150981

Life-Threatening Sochi Virus Infections, Russia

Technical Appendix

Technical Appendix Table 1. Details of serodiagnostic data (reciprocal antibody titers) of the 62 patients*

Patient	No.	Sampling day	IFA				ELISA				FRNT					
	serum	after disease					PUUV		DOBV			DOBV				
no.	samples	onset	PUUV	DOBV	HTNV	SEOV	IgM	IgG	IgM	IgG	PUUV	Sochi	Kurkino	Dobrava	HTNV	SEOV
1	1,293	27	<16	1024	1024	512	<128	<128	16384	32768	<40	1280	640	640		
	1312	104	<16	2048	512	512	<128	<128	2048	8192	<40	1280	160	160	160	<80
2	1308	22	<16	2048	1024	1024	256	<128	4096	4096	<40	640	80		160	80
	1310	50	<16	8192	4096	4096					<40	1280	640	640	160	160
	4708†	5 y 9 m	64	2048	2048	2048					<40	2560	1280	2560	80	40
3	1307	15	<16	4096	2048	2048	<128	<128	8196	8196	<40	640	320	320	160	160
4	1335	17	64	8192	4096	4096	512	512	8192	16384	<40	1280	<80	80		
	1336	82	<32	4096	2048	1024	1024	512	4096	32768	<40	640	320	640	<80	160
	4,709†	5 y 9 m	64	4096	4096	2048					<40	5120	1280	2560	160	80
5	1339	11	<32	4096	2048	2048	256	<128	8192	8192	<40	320	80			
6	1642	3	<16	2048	1024	512	<128	<128	8196	2048						
	4716†	5 y 3 m	128	4096	4096	4096					<40	5120	1280	2560	160	160
7	1686	16	<16	2048	1024	512	<128	<128	2048	2048	<40	640	80	80		
8	4712	9	<16	512	256	256	<128	<128	2048	512						
9	3507	15	64	4096	2048	2048	<128	<128	8196	8196	<40	640	160	320		
	4713†	1 y 5 m	1024	8192	4095	4096					<40	20480	5120	10240	320	160
10	3830	13	128	8192	2048	2048	512	256	16384	8192	<40	640	160	640		
	4714†	1y 5m	512	16384	16384	8192					<40	20480	5120	5120	80	40
11	3692	30	64	4096	4096	2048	256	256	4096	16384	<40	1280	160	<160		
12	3693	10	32	2048	1024	1024	<128	<128	16384	8192						
	3694	18	64	2048	2048	2048					<40	640	<80	80		
13	3496	9	<16	4096	4096	2048	<128	<128	16384	16384						
14	3802	11	64	4096	2048	2048	<128	<128	8192	2048	<40	1280	320	640		
	4711†	1 y 6 m	512	8192	8192	4096					<40	20480	20480	20480	160	80
15	3778	10	64	8192	4096	4096	<128	<128	65536	8192						
16	3824	11	64	8192	8192	4096	<128	<128	16384	16384						
17	3813	4	64	8192	2048	2048	<128	<128	16384	16384						
	4710†	1 y 5 m	64	2048	2048	2048					<40	10240	5120	5120		
18	3861	16	256	8192	4096	4096	<128	<128	8192	2048	<40	640	640	640		

Patient no.	No. serum samples	Sampling day after disease onset	IFA				ELISA				FRNT					
							PUUV		DOBV			DOBV				
			PUUV	DOBV	HTNV	SEOV	IgM	IgG	IgM	IgG	PUUV	Sochi	Kurkino	Dobrava	HTNV	SEOV
	4715†	1 y 4 m	128	8192	8192	4096					<40	5120	2560	5120	80	40
19	3855	9	<16	4096	2048	2048	<128	<128	16384	2048						
20	4387	8	<16	512	256	256	<128	<128	1024	1024						
21	4471	2	<16	512	512	256	<128	<128	4096	1024						
22	4705	?	128	2048	2048	2048	128	<128	4096	4096	<40	640	640	640		
23	5049	7	<16	1024	512	512	<128	<128	4096	1024						
24	5065	8	<16	2048	2048	2048	<128	<128	8192	8192						
25	5066	8	<16	2048	1024	512	<128	<128	4096	8192						
26	5067	8	<16	1024	512	512	<128	<128	4096	1024						
27	5293	12	<16	4096	2048	2048	<128	<128	8192	8192						
28	5312	5	<16	1024	1024	1024	<128	<128	4096	1024						
29	6624	4	<16	4096	2048	2048	<128	<128	16384	4096						
30	6625	6	64	2048	2048	2048	<128	<128	4096	1024						
31	6627	7	64	4096	1024	1024	<128	<128	8192	8192						
	6627-2	73	64	4096	512	512					<40	2560	320	640	160	80
32	6623	13	32	4096	4096	2048	128	128	16384	8192						
33	6626-2	108	256	8192	2048	2048					<40	10240	1280	2560	160	160
34	6930	5	256	4096	2048	2048	<128	<128	8192	8192						
35	6845	4	<16	1024	512	256	<128	<128	8192	4096						
	6845-a	29	<32	1024	512	512					<40	1280	160	320	<40	<40
	6855-2	11 m	512	4096	1024	1024					<40	2560	640	640	80	80
36	6931	7	64	2048	512	512	<128	<128	4096	8192						
37	6918	13	<32	2048	1024	512	<128	<128	8192	16384						
	6918a	27	<32	2048	1024	512					<40	2560	320	1280	40	<40
	6931-2	1y	512	4096	1024	1024					<40	5120	1280	2560	160	160
38	4481	2 m	256	8192	8192	4096	<128	<128	2048	16384		2560	640	640	160	160
40	4482	9	512	8192	4096	2048	<128	<128	16384	16384	<40	2560	2560	2560	<40	<40
41	4483	9	1024	16384	8192	8192	512	256	16384	16384						
42	4484	15	64	8192	2048	2048	<128	<128	16384	32768	<40	320	80	80	<40	<40
43	4485	9	128	8192	4096	4096	256	256	32768	32768						
44		12	512	8192	4096	4096	512	512	16384	16384						
45	6876	6	<16	4096	4096	4096	<128	<128	16384	8192						
46	6878	11	1024	32000	4096	4096	512	256	32768	32768						
	6878-a	33	64	8192	4096	1024					<40	5120	640	2560	160	80
47	6880	3	<16	4096	2048	2048	<128	<128	4096	1024						
48	6929	4	<16	4096	4096	4096	<128	<128	16384	1024						
49	6628	19	<32	2048	1024	1024	<128	<128	4096	8192						
	6875	26	<32	1024	512	512					<40	2560	640	640	160	160
51	6882	17	64	4096	2048	2048	256	<128	8192	8192						

Patient no.	No. serum samples	Sampling day after disease onset	IFA				ELISA				FRNT					
							PUUV		DOBV			DOBV				
			PUUV	DOBV	HTNV	SEOV	IgM	IgG	IgM	IgG	PUUV	Sochi	Kurkino	Dobrava	HTNV	SEOV
	6882-a	34	64	4096	2048	1024					<40	1280	320	640	40	<40
52	6883	13	256	2048	1024	1024	<128	<128	8192	8192						
	6883-2	10 m	256	4096	2048	512					<40	2560	80	160	40	40
53	7105	10	256	4096	4096	2048	256	128	32768	32768						
54	7110	9	64	1024	1024	1024	<128	<128	2048	4096						
55	7111	5	64	1024	1024	512	<128	<128	8192	4096						
56	8385	4	<16	1024	512	512	<128	<128	8192	2048						
57	8386	8	64	1024	1024	512	256	128	4096	8192						
58	8380	6	32	2048	1024	1024	<128	<128	8192	8192						
59	8381	5	<16	128	64	64	<128	<128	1024	128						
60	8382	9	32	4096	4096	1024	<128	<128	32768	16384						
61	8383	16	<16	4096	4096	2048	<128	<128	16384	16384						
62	8384	24	64	4096	4096	2048	256	256	8192	32768						
63	8388	5	<16	4096	2048	2048	<128	<128	8192	8192						

*Patient serum samples were screened for hantavirus antibodies by indirect IFAs by using spot-slides containing a mixture of Vero-E6 cells infected by PUUV, DOBV, HTNV, and SEOV, as previously described (Dzagurova et al., Zh Mikrobiol Epidemiol Immunobiol, 2008; No.1: 12-16). Serum found to be positive was confirmed and further typed on IFA with “monovalent” spot-slides containing cells infected by only 1 of the mentioned viruses. Thereafter, IgG and IgM were determined by ELISAs on the basis of PUUV and DOBV antigens by established methods (Meisel et al., Clin Vaccine Immunol, 2006;13: 1349-57). For further serotyping, the neutralizing activity of serum was investigated by FRNT (Dzagurova et al., Zh Mikrobiol Epidemiol Immunobiol, 2008; No.1: 12-16), using Vero-E6 cells to propagate the following virus stocks; PUUV strain K-27/Ufa-85, DOBV (genotype Sochi) strain Ap1584/Sochi-01 (named Sochi/Ap), DOBV (genotype Kurkino) strain Aa1854/Lipetsk-02, DOBV (genotype Dobrava) strain Bel-1, HTNV strain P-88/ Khabarovsk-89, SEOV strain SR-11. DOBV, Dobrava-Belgrade virus; FRNT, focus-reduction neutralization assay; HTNV, Hantaan virus; IFA, immunofluorescence assay; PUUV, Puumala virus; SEOV, Seoul virus. Blank cells indicate testing not done.

†FRNT was performed with addition of 10% guinea pig blood serum (as complement).

Technical Appendix Table 2. Virus sequences and corresponding GenBank accession numbers used in the phylogenetic analyses

			GenBank accession no.	
Virus species	Abbreviation	Strain name	S segment	L segment
Dobrava-Belgrade virus	DOBV	10636/Ap	**KP878311**	**KP878308**
		10645/Ap	**KP878312**	**KP878309**
		10752/hu	**KP878313**	**KP878310**
		6882/hu	–	**KM192207**
		43/Ap	JF920151	**KM192209**
		79/Ap	JF920152	**KM192208**
		Sochi/hu	JF920150	JF920148
		Sochi/Ap	EU188449	EU188451
		GK/Ap	AF442622	–
		Krasnodar/hu	AF442623	–
		SK/Aa	AY961615	GU904039
		Esl29/Aa	AY533118	–
		Esl81/Aa	AY533120	–
		Kurk44/Aa	AJ131672	–
		Kurk/53/Aa	AJ131673	–
		Esl856/Aa	AJ269549	–
		Esl862/Aa	AJ269550	–
		Lipetsk/Aa	EU188452	EU188454
		GER/08/118/Aa	GQ205407	–
		GER/08/131/Af	GQ205408	–
		GER/07/293/Aa	GQ205401	–
		GER/07/607/Af	GQ205402	–
		GER/07/1064/Aa	GQ205404	–
		GER/05/239/Aa	GQ205405	–
		GER/05/477/Af	GQ205406	–
		Esl400/Af	AY168576	–
		AP9/Af	AJ410615	AJ410617
		AP13/Af	AJ410619	–
		Slo/Af	L41916	AJ009779
		Istanbul/H56	–	KF039740
		Saa/160V	AJ009773	AJ410618
		Saa/90Aa	AJ009775	–
Sangassou virus	SANGV	SA14	JQ082300	JQ082302
		SA22	JQ082303	–
Hantaan virus	HTNV	76–118	M14626	NC_005222
		SC-2	–	AY675354
		Z10	AF184987	–
		LR1	AF288294	–
		AH09	AF285264	–
		84FLi	AY017064	–
Thailand virus	THAIV	741	AB186420	–
		NR/Bi0017	AM397664	–
Seoul virus	SEOV	80–39	NC 005236	NC_005238
		Gou3	AF184988	–
		Z37	AF187082	–
		L99	AF488708	–
Puumala virus	PUUV	CG1820	–	M63194
Tula virus	TULV	Mo5302	–	NC_005226
Andes virus	ANDV	Chile-9717869	–	NC_003468
Sin Nombre virus	SNV	NM H10	–	NC_005217

*The nucleotide sequences generated in this study were based on PCRs amplifying a 390-nt conserved region within the L segment (Klempa et al. Emerg Infect Dis. 2006;12:838–40) or a 599-nt fragment of the S segment (Klempa et al., J Clin Microbiol, 2005;43:2756–63). Bold type indicates new sequences. Dashes indicate that no sequence information was available.

Technical Appendix Table 3. Classification criteria of clinical severity of hemorrhagic fever with renal syndrome*

	Clinical course		
Symptoms	Mild	Moderate	Severe
Duration of fever, d	3–4	5–6	>6
Systolic blood pressure, mm Hg	100	<100	<80
Hemorrhagic syndrome	Scleral, subcutaneous hemorrhages	Bleedings, not life threatening	Bleedings, life threatening
Duration of oliguria <500 mL/24 h	24–48 h	49-96 h	>96 h
Anuria >50 mL/24 h	–	–	Positive
Serum creatinine, µmol/L	Normal (<96/F, <110/M)	120–250	>250
Proteinuria, g/L	<1	<5	>5
Pulmonary edema	–	–	Positive
Cerebral edema	–	–	Positive
Kidney rupture	–	–	Positive

*Classification according to Russian standard criteria (Leshchinskaia et al., Vopr Virusol. 1990;35:42–5; Klempa et al., Emerg Infect Dis.2008;14:617–25). Dashes indicate that these symptoms were not observed.
